# Oxidative Stress in Placenta: Health and Diseases

**DOI:** 10.1155/2015/293271

**Published:** 2015-11-29

**Authors:** Fan Wu, Fu-Ju Tian, Yi Lin

**Affiliations:** Institute of Embryo-Fetal Original Adult Disease, The International Peace Maternity & Child Health Hospital, Shanghai Jiao Tong University School of Medicine, Shanghai 200030, China

## Abstract

During pregnancy, development of the placenta is interrelated with the oxygen concentration. Embryo development takes place in a low oxygen environment until the beginning of the second trimester when large amounts of oxygen are conveyed to meet the growth requirements. High metabolism and oxidative stress are common in the placenta. Reactive oxidative species sometimes harm placental development, but they are also reported to regulate gene transcription and downstream activities such as trophoblast proliferation, invasion, and angiogenesis. Autophagy and apoptosis are two crucial, interconnected processes in the placenta that are often influenced by oxidative stress. The proper interactions between them play an important role in placental homeostasis. However, an imbalance between the protective and destructive mechanisms of autophagy and apoptosis seems to be linked with pregnancy-related disorders such as miscarriage, preeclampsia, and intrauterine growth restriction. Thus, potential therapies to hold oxidative stress in leash, promote placentation, and avoid unwanted apoptosis are discussed.

## 1. Introduction

Oxidative stress (OS) is broadly referred to as an imbalance between the generation of reactive oxygen species (ROS) or reactive nitrogen species (RNS) and their clearance by defensive antioxidants [1]. Superoxide radicals (O_2_^∙−^), hydroxyl radicals (HO^∙^), hydrogen peroxide (H_2_O_2_), peroxynitrite (ONOO^−^), and nitric oxide (NO) are common oxygen-derived ROS and RNS [2]. They are frequently generated in the placenta by the mitochondrial respiratory chain and prooxidative enzymes like xanthine oxidase (XO) and NADPH oxidase (Nox) [3–5]. Excessive OS are generally thought to be involved in the pathology of many pregnancy-related disorders. Premature maternal-fetal circulation and widespread blood OS attack lead to extensive placental injury and are potential causes of first-trimester spontaneous abortion [6, 7]. However, insufficient placental perfusion and ischemia/reperfusion (I/R) induced OS are associated with preeclampsia (PE) and intrauterine growth restriction (IUGR) [8, 9]. The benefits of well-controlled ROS and RNS are gradually being recognized. These reactive species are involved in many important cellular signaling pathways and induce the expression of physiologically necessary genes [10]. NO from endothelial nitric oxide synthase (eNOS) has multiple functions including vasodilatation, anti-inflammation, antithrombosis, and proangiogenesis [11]. More and more compounds have been investigated to reverse OS conditions and promote placentation without interfering with biological markers in normal OS-related signaling.

## 2. Oxidative Stress and Placenta Formation

The proper development of the trophoblast lineage and uterine vessels is a key precondition for human successful pregnancy. Initially, the blastosphere is encircled by a thin layer of mononucleated cytotrophoblasts (CTBs) [12]. Once attached to the endometrium, these cells rapidly proliferate, and the outer layer fuses to form multinucleated syncytiotrophoblasts (STBs), while an inner cluster becomes invasive extravillous trophoblasts (EVTs) which soon spread out into the uterine stroma [12]. According to “two-wave invasion” theory, this type of invasion may be relatively preliminary within decidual layer and is followed by a pause until around week 12 of human gestation when a second wave of deep and diffuse invasion begins [12]. In this wave of invasion, EVTs widespreadly infiltrate into the endometrium and part of the myometrium as well as efficiently expand maternal spiral arteries [12]. Both the interstitial and endovascular penetration events are indispensable to keep the fetus rooted and enable large-caliber, low-resistance maternal-fetal circulation [12, 13]. A deficiency in trophoblast invasion, especially the second wave, is associated with PE and IUGR. It could be attributed to activation of mitochondria, XO, and Noxs by I/R [8]. A mass of ROS from these sources inactivates biomacromolecules and disrupts cellular metabolism, leading to endothelial dysfunction and excessive trophoblast apoptosis as well as increasing anti-angiogenic soluble fms-like tyrosine kinase-1 (sFlt-1) and soluble endoglin (sEng), which bind and neutralize circulating proangiogenic vascular endothelial growth factor (VEGF) and transforming growth factor-*β*1 (TGF-*β*1), respectively [4, 8, 9, 13].

With the progression of trophoblast differentiation and maturation of blood supply, the placental oxygen concentration also changes. Before week 12 of human gestation, the placental intervillous space is actually low oxygen. Morphological examinations and Doppler ultrasound inspections have shown that during this time the placenta is not hemochorial, and trophoblast cells plug the tips of the uteroplacental arteries [14, 15]. Around the end of the first trimester, these plugs gradually vanish accompanied by the second wave of trophoblast invasion, and free-flowing blood causes a significantly steep increase of placental oxygen partial pressure from <20 mmHg (2–4%) at week 8 to >50 mmHg (10%) at week 12, triggering enhanced OS in trophoblasts [6]. Notably, the entrance of blood and the OS originate in the periphery and extend progressively to the rest of the placenta [7, 13]. This step-by-step periphery-to-center spread of OS is conducive to the degradation of the chorion frondosum to form the chorion laeve [7, 13]. At the same time, it protects the fetus from a sudden OS increase, since an untimely and premature entry of a large amount of maternal blood OS may be one of the reasons leading to early pregnancy failure [6, 7]. As placental OS may closely interact with vascular and trophoblastic biological properties, we will further discuss how placental growth and OS fluctuation accommodate and affect each other.

### 2.1. Angiogenesis and Vasculopathy

VEGF and placenta growth factor (PlGF) are well known to play crucial roles in endothelium growth, albeit during different times and in different ways [16]. ROS from chronic hypoperfusion and low oxygen in the first trimester drive the expression of VEGF through hypoxia inducible factor-1 (HIF-1), a transcription factor adapting to hypoxidosis, yet high oxygen later downregulates VEGF [17]. PlGF appears to be regulated in the opposite direction, being present at a low level during low oxygen and increasing along with the elevation of oxygen concentration [16]. Accordingly, VEGF has been demonstrated to be a potent stimulator of endothelial proliferation and migration to support branching angiogenesis in the first trimester, while PlGF is thought to promote nonbranching angiogenesis in the second and third trimesters [16]. Thus, premature hemoperfusion and hyperoxia in early gestation may lead to reduced VEGF levels and a premature PlGF peak, potentially causing maldevelopment of villous vessels and pregnancy failure [16, 17]. ROS also serve as crucial signaling molecules. VEGF and Angiopoietin-1 (Ang-1) can stimulate ROS generation through different endothelial Nox isoforms that are involved in VEGF receptor-2 phosphorylation, as well as stimulation of downstream extracellular regulated protein kinase-1/2 (ERK-1/2), Akt, and eNOS [18–21]. Various transcription factors, including HIF-1 and others, closely associated with OS take part in vascular development [20–22].

OS is also able to promote angiogenesis through growth factor-independent pathways. ROS can oxidize phospholipids to produce carboxyalkyl pyrroles (CAPs) [23]. CAP protein adducts are endogenous ligands for toll-like receptors (TLRs) such as TLR2 on endothelial cells that induce neovascularization [23]. Another mediator of OS-induced angiogenesis is ataxia-telangiectasia mutated (ATM) kinase, which was shown to promote only pathological neoangiogenesis [24].

Enhanced OS with insufficient scavenging systems can result in vascular aging and diseases [25]. Noxs may participate in the pathogenesis of atherosclerosis and hypertension and are therefore a hopeful drug target to rescue abnormal vessel development [26]. Peroxidized lipids are also linked to atherogenic processes and pathological angiogenesis [27]. In IUGR, a vascular process similar to atherosclerosis was observed in the placenta, presenting as reduced arterial diameter and increased plasma OS biomarkers [28]. OS is implicated in directly oxidating NO to ONOO^−^ that reduce NO availability, rendering vessels prone to constriction, inflammation, and thrombosis [29]. Furthermore, OS induce the uncoupling of eNOS by oxidating NOS cofactor tetrahydrobiopterin (BH4) and eNOS uncoupling generates O_2_^∙−^ and ONOO^−^ that further decrease NO bioavailability [29].

### 2.2. Proliferation, Differentiation, and Invasion of Trophoblasts

Genbacev et al. observed an interesting phenomenon in which some CTBs incubated in low oxygen tension mainly proliferated, showing poor ability to differentiate and invade, while, at high oxygen concentration, cells stopped proliferating and differentiated normally [30]. This finding suggests the* in vivo* alteration of trophoblast cells. In the first trimester, CTBs in low oxygen may show strong characteristics of proliferation but weak capacities for invasion and differentiation [6, 12, 13, 31]. Later, a burst of OS may switch CTBs from a proliferative phenotype to the invasive extravillous phenotype that is required for the secondary wave of trophoblast invasion [6, 12, 13, 31].

Analyses of the underlying mechanisms have demonstrated that OS changes the repertoire of integrins. Hypoxia could inhibit the expression of CTB *α*1/*β*1 integrins, while it upregulates *α*5 or *α*6/*β*1 integrin subunits, thus broadly inhibiting the conversion of CTBs to the extravillous phenotype while enhancing placental growth [32, 33]. Hypoxia also inhibits the activation of matrix metalloproteinases (MMPs) such as MMP-2 in EVT cells [34], while a well-oxygenated environment leads to an increase of EVT cell invasion by increasing activation of *α*1 integrins, MMP-2, and MMP-9 [34, 35].

HIF-1 is thought to participate together with TGF-*β*3 in the regulation of MMPs and integrins [33, 36]. In response to low oxygen, HIF-1 promotes CTB proliferation but blocks differentiation and invasion [31, 36, 37]. When the placental O_2_ level increases, downregulated HIF-1 subsequently decreases TGF-*β*3 level, which in turn leads to the activation of MMPs and the shift in integrin isoforms [33, 36, 37]. Studies have suggested that, in PE, trophoblasts do not express normal levels of integrin *α*1 and MMP-9, which impedes EVT invasion and blood perfusion of the placenta [37, 38].

CTB cells have the capability of fusing to become STBs, which play vital roles in the material exchange and hormone synthesis necessary for fetal development [39]. Overexpression of superoxide dismutase (SOD) has been shown to restrain the ability of CTBs to fuse and differentiate into STBs, accompanied by a significant decrease in expression of hormones such as human chorionic gonadotrophin (hCG), human placental lactogen (hPL), and placental growth hormone (pGH), all of which are indicators of properly differentiated STBs [39]. Examination of placenta tissues from pregnancies with PE or IUGR has revealed an intrinsic reduction in cell fusion, leading to increased apoptosis [40]. Attention on the mechanism of CTB fusion and its potential relationship with redox system may further help to understand the etiology of abnormal placentation.

## 3. Interaction between Autophagy and OS

**Figure 1:**
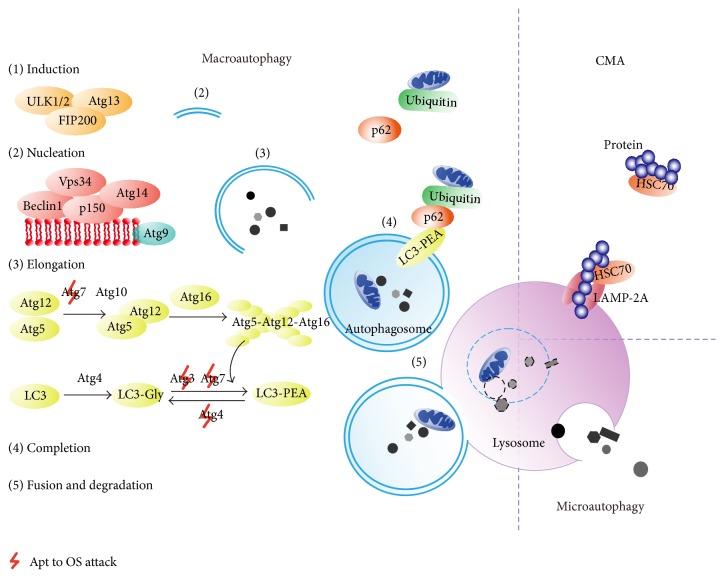
Mechanisms of autophagy. In macroautophagy, the phagophore is induced by the ULK1/2-Atg13-FIP200 (focal adhesion kinase family-interacting protein of 200 kDa) complex and nucleated by class III phosphatidylinositol 3-kinase (PtdIns3K) complex, which is composed of Vps34 (vacuolar protein sorting 34), Beclin1, p150, and Atg14. Thereafter, Atg4 interacts with LC3 (the microtubule-associated protein light chain 3) to form LC3-I (LC3-Gly) [42, 46, 47]. The Atg12-Atg5-Atg16 complex together with Atg3 and Atg7 stimulates LC3-I to bind with phosphatidylethanolamine (PEA) to produce the LC3-PEA complex (LC3-II), during which the autophagosomal membrane begins to extend and enclose to form an integrated autophagosome [42, 46, 47]. Atg9, like a transport cart, circulates to carry membrane materials for the elongation and expansion of the autophagosomal vesicle [46, 47]. In the end, the mature autophagosome docks and fuses with the lysosome where all of its contents are degraded by acid hydrolases [42, 46, 47]. Macroautophagy can sometimes selectively clear ubiquitinated proteins linked with p62, since p62 works with LC3-II to entrap these long-lived proteins into autophagosomes [48]. In other types of autophagy, such as chaperone-mediated autophagy (CMA), lysosomes selectively degrade cytoplasmic proteins with the KFERQ-related motif, which can be recognized by chaperone HSC70 (heat shock cognate protein 70) [42]. LAMP-2A (lysosome-associated membrane protein 2A) then mediates their entry into lysosomes [42].

Autophagy, which has been a research hot-topic in recent years as an important self-adjusting catabolic process to remove redundant or damaged organelles and proteins in the regulation of lysosomes [41], is categorized into three types [42]. Macroautophagy, hereafter referred to simply as autophagy, is the most common and best studied. It relies on a double-membraned vacuole (autophagosome) to parcel relatively large volumes of cellular constituents; the autophagosome then combines with lysosomes to eliminate the cellular material (Figure 1) [41, 42]. The other two types are chaperone-mediated autophagy (CMA) and microautophagy (Figure 1) [42]. According to many studies, O_2_^∙−^, HO^∙^, and H_2_O_2_ released by the mitochondria or produced by reducing agents are all considered to stimulate autophagy [41, 43–45].

**Figure 2:**
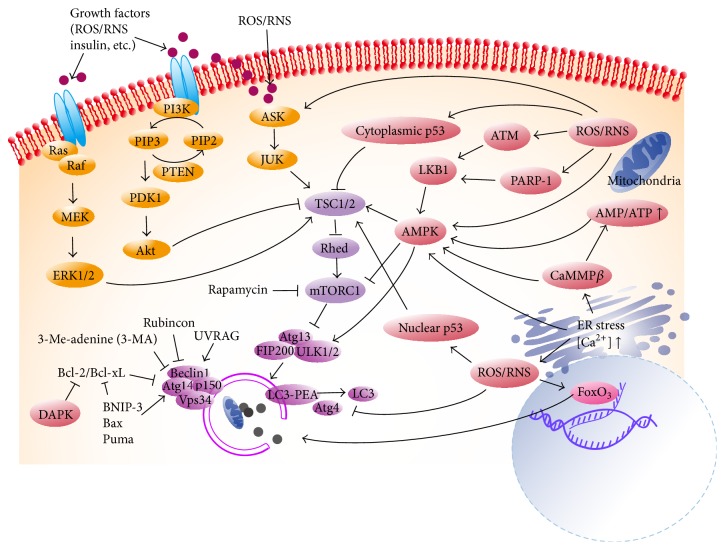
Autophagic signaling pathways associated with oxidative stress. External OS and other stimuli can activate the PI3K-Akt-mTORC1, Ras-MEK-ERK1/2, and ASK- JUK axis [42, 49, 50]. Activated PI3K recruits PDK1 (phosphoinositide-dependent kinase 1) and phosphorylates Akt, inactivating the TSC1/2 (tuberous sclerosis complex 1/2) and leading to mTORC1 activation [42]. mTORC1 is an energy/redox sensor, the function of which can be blocked by rapamycin [42]. It represses autophagy by phosphorylating Atg13 and separating it from the ULK kinase complex [42]. Elevated cytoplasmic ROS/RNS can be sensed by AMPK, ATM kinase, and PARP-1 [47, 51–53]. AMPK activates autophagy by inhibiting mammalian target of rapamycin complex 1 (mTORC1) and directly activating Unc-51-like kinase 1/2 (ULK1/2) [42, 51]. ATM or PARP-1 are able to activate the LKB1 (liver kinase B1)-AMPK-TSC1/2 metabolic pathway to repress mTORC1 or directly activate ULK1/2 through phosphorylation of its serine groups, upregulating autophagy [52, 53]. As well as OS levels, AMPK also responds to cellular AMP/ATP levels, endoplasmic reticulum (ER) stress, and CaMKK*β* (calmodulin-dependent protein kinase kinase-*β*)-related signaling [42, 46, 51]. p53 exhibits paradoxical autophagic regulation in OS, as nuclear p53 positively enhances autophagy through TSC1/2-dependent pathways, whereas cytoplasmic p53 seems to do the opposite [98]. DAPK, BNIP, Bax, and Puma all function to break down the interaction between Bcl-2/Bcl-xL and Beclin-1, normalizing the essential formation of the class III PtdIns3K complex [64, 101, 102]. UVRAG (ultraviolet irradiation resistance-associated gene) also positively regulates this PtdIns3K complex, while Rubicon counteracts its effect [42].

In mammalian cells, autophagy is a complicated process requiring many autophagy-related (Atg) genes and proteins (Figure 1) [46–48]. Extracellular stress regulates autophagy through phosphatidylinositol 3-kinase- (PI3K-) Akt pathways and mitogen-activated protein kinases (MAPKs) like ERK and Jun N-terminal kinase (JUK) (Figure 2) [42, 49, 50]. In the cytoplasm, AMP-activated protein kinase (AMPK), ATM kinase, and poly(ADP-ribose) polymerase-1 (PARP-1) have been reported to stimulate autophagy in response to OS or OS-related damage, and the results can be either helpful or destructive (Figure 2) [47, 51–53]. Autophagy-mediated cell death has been shown in some experiments [53, 54]. Cellular death associated with autophagy is defined as type II cell death, which is distinguished from apoptosis (type I) and necrosis (type III) [47]. However, other data show that autophagy plays a cytoprotective role in H_2_O_2_-induced cell death [52]. As for transcription factors, forkhead box O (FoxO) is a well-known “peacemaker” protein that is induced by OS and enhances antioxidant activity and inhibits apoptosis [55]. FoxO has also been demonstrated to promote expression of* Atg* genes to resist OS attack [56]. Therefore, FoxOs link autophagy with other cellular mechanisms [55, 56]. The conjugation of LC3-I and phosphatidylethanolamine (PEA) is essential to autophagosomal formation, while Atg4 protease works to dissociate them [46, 47]. OS can render Atg4 inactive by oxidizing residue cysteine-81, thereby promoting lipidation of LC3-I, which is conducive to autophagy [57]. Other cysteine-containing proteins such as Atg3 and Atg7 may also be disabled by OS, resulting in inhibition of autophagy [58]. Autophagy, in turn, influences redox signaling pathways [59, 60]. Deficiency in autophagy has been shown to cause the accumulation of p62 [59]. Excessive p62 activates nuclear factor *κ* B (NF-*κ*B) pathway, which leads to suppression of OS [59]. Moreover, the build-up of p62 activates nuclear factor-erythroid 2 related factor 2 (Nrf-2) and its target antioxidant genes [60].

Autophagy seems to be induced in trophoblasts from early gestation [61].* In vitro* experiment showed that oxygen deficit evokes autophagy in primary human trophoblast cells [62]. In normoxia, autophagy is thought to be kept at a low level as Beclin1 (the mammalian ortholog of the yeast Atg6 gene) binds with low affinity to B-cell lymphoma-extra-large (Bcl-xL) and B-cell chronic lymphocytic leukemia/lymphoma 2 (Bcl-2) via its Bcl-2 homology 3 (BH3) domain [63, 64]. In the first trimester, low oxygen induces HIF-1, which then may initiate the expression of proapoptotic Bcl-2/adenovirus E1B 19 kDa interacting protein 3 (BNIP-3), a BH3-only protein that can separate Beclin1 from Bcl-xL and Bcl-2, activating autophagy [63, 64]. This kind of basal autophagy may function to clear undesired proteins and damaged organelles. Without autophagic scavenging, damaged mitochondria recruit monocyte chemotactic protein-1 (MCP-1), interleukin-6 (IL-6), and IL-1*β*, resulting in systematic inflammation [65]. Autophagy also allows for recycling of nutrients for reuse in an energy-saving way [66]. Thus, autophagy may be important to protect trophoblast cells in the oxygen-insufficient, nutrient-limited environment of the early placenta. Further it seems to limit the effect of proapoptotic proteins like BNIP-3 induced by low oxygen [63]. However, beyond a certain limit, autophagy may destroy cellular structures, breaking down energy supplies and initiating autophagic cell death [54]. In some conditions, autophagy may selectively degrade ROS scavengers such as catalase, making it hard to contain the damage from OS [67]. In short, ROS/RNS-related autophagy is a double-edged sword, which varies in its intensity and is dependent on the cell type [66].

Mitochondria are one of the major sources of ROS in trophoblast cells and also the targets of increasing OS. Damaged mitochondria not only produce larger amount of ROS but also release proapoptotic factors like cytochrome* c *[68]. Also, mitochondria show dynamic fusion and fission activities as one of mitochondrial quality control pathways [68]. Fission can produce impaired or depolarized mitochondria and segregate them from the whole mitochondrial population [68]. A special pattern of autophagy called “mitophagy” thus works to degrade these damaged, dysfunctional, or superfluous mitochondria to maintain normal mitochondria turnover and cell homeostasis [68]. Nonselective autophagy like that activated by AMPK could induce mitophagy [69]. Many studies showed that mitophagy may also be a selective way. If mitochondria are impaired or loss of membrane potential, PTEN-induced putative protein kinase 1 (PINK1) could accumulate on mitochondrial outer membrane in time [70, 71]. As a result, PINK1 recruits Parkin from the cytosol to these damaged mitochondria where Parkin ubiquitinates various membrane proteins [70, 71]. p62 then may recognize the ubiquitinated proteins and guide mitochondria into autophagosomes through interacting with LC3 (Figure 1) [72]. However, a study showed that p62 may not be the requisite adaptor in the PINK1/Parkin-mediated mitophagy and p62 may work for aggregating ubiquitinated damaged mitochondria yet have little effect on mitochondrial degradation [73]. As to receptor-mediated mitophagy, mitochondrial protein Nix/BNIP3L is a selective autophagy receptor that could bind to LC3/GABARAP and induce the formation of autophagosomal membranes directly, although it has the ability to activate autophagy by releasing Beclin-1 from Bcl-2 [74, 75]. Nix/BNIP3L also participate in mitochondrial priming by promoting Parkin translocation to the mitochondrial membrane [75]. Other mitochondrial receptors like BNIP-3 and FUNDC1 similarly have a classic motif to directly bind LC3 to induce mitophagy [76, 77].

Autophagy is also reported to play a role in EVT invasion and vascular remodeling at a physiologically low oxygen concentration, suggesting a contribution of autophagy to normal placentation [78, 79]. Autophagy impaired by sEng may partially be related to poor placentation in PE due to suppressed EVT invasion and poor vascular remodeling [79]. Replacement of endothelial cells by EVT is impaired by disruption of autophagy [79].

However, other data suggest that increased autophagic activity in CTB and STB may be a negative factor in the pathophysiology of PE and IUGR [80–82]. It is confused when we talk about the exact role of autophagy in placenta. Some of these experiments just test the increasing autophagy associated proteins in pathological human pregnancy while they did not proven whether autophagy is the cause or just an accompanying phenomenon of other mechanism. Additionally, higher apoptosis in PE and IUGR than normal placenta is a wider acceptance and autophagy is closely related to apoptosis which we will discuss next.

## 4. Apoptosis Induced by OS

Apoptosis, or programmed cell death, is a physiological phenomenon in trophoblast turnover [83]. However, increased apoptotic markers are observed in pathological PE placentas and in patients with hemolysis elevated liver enzymes and low platelets (HELLP) syndrome and IUGR [83, 84]. OS can induce apoptosis via external or intrinsic signals. The former are mediated by cell-surface death receptor Fas-induced caspase-8, while the latter are transmitted by mitochondria-mediated caspase-9 pathways [83, 85]. The two signaling pathways are not completely separate as they converge on the activation of caspase-3, resulting in apoptotic-like chromatin condensation and cell shrinkage [85]. OS could also modify some key apoptotic regulators, such as proteins of the Bcl-2 family, p53, and components like apoptosis signal-regulating kinase-1 (ASK-1), c-JNK, and p38 MAPK [85–87].

ASK1 is normally kept inactive by attachment to thioredoxin, but ROS are able to oxidize the thiol groups in this binding protein, releasing ASK1 to become dimerized and phosphorylated [2]. The activated ASK1 then stimulates the downstream family of MAPKs [87]. Within this family, the activation of ERK-1/2 generally promotes cell survival and proliferation, whereas stimulation of p38 and c-JNK mostly induces apoptosis and injury [87].

p53 is an important sensor of OS, and p53 expression may further trigger OS [88]. In response to OS stimuli, p53 induces expression of downstream elements such as cell cycle inhibitor p21 and caspase activator apoptotic protease activating factor 1 (APAF1) [89]. In normal circumstances, cellular p53 is restrained by murine double minute 2 (Mdm2), which has an E3-like function to remove p53 so that pro- and antiapoptotic proteins are kept in balance [89, 90]. In placentas with PE, however, significantly increased p53, p21, and Bax and conversely exhausted Mdm2 may lead to excessive apoptosis and placental malfunctions [89].

Bcl-2 family proteins play a major role in the intrinsic pathway of OS-related apoptosis. Myeloid cell leukemia factor-1 (Mcl-1) is characterized by its antiapoptotic and antiproliferative effects, while matador/Bcl-2 ovarian killer (Mtd/Bok) favors cell proliferation and at elevated levels facilitates apoptosis [91, 92]. Since low oxygen during early placentation promotes both Mtd-L (L: long isoform) and Mcl-1L production, the dynamic balance of death-inducing Mtd-L and antiapoptotic Mcl-1L is delicate in maintaining trophoblast homeostasis [92]. In PE or I/R, however, Mcl-1L is caspase-dependently cleaved to become the death-inducing isoform Mcl-1S, losing the ability to neutralize Mtd-L [91]. At the same time, a decrease in the amount of Mcl-1L is accompanied by relative overexpression of Mtd/Bok in PE, disturbing the management of the Mtd/Bok-Mcl-1 rheostat [91]. The result may be a hyperproliferative phenotype or excessive trophoblast apoptosis [93]. The Mcl-1 ubiquitin ligase E3 (MULE) is another regulator of Mcl-1, yet its mechanism is more complex since p53 is also a target of MULE [94]. In PE, MULE preferentially clears p53, making proapoptotic Mcl-1 isoforms dominant [94]. In IUGR, however, MULE changes its role to scavenge prosurvival Mcl-1 isoforms, leaving p53 to exert its apoptotic effect [94]. The different and intermingled regulatory processes reveal that different molecular mechanisms may underlie different complications, and their counterbalance may be a basis of adaptive plasticity.

**Figure 3:**
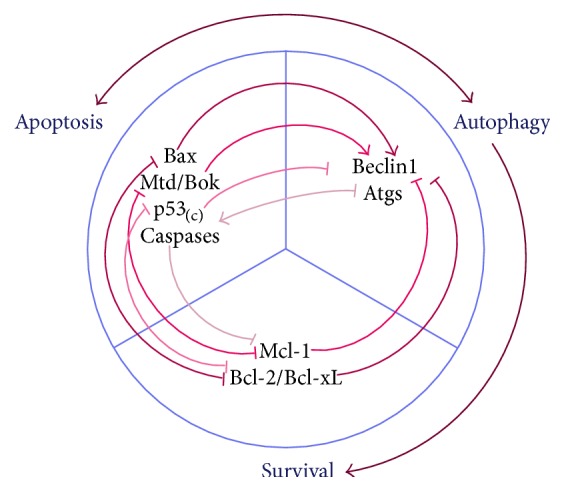
Crosstalk between autophagy and apoptosis. Many lines of evidence have proven the antiautophagic role of apoptosis. Cytoplasmic p53 inhibits the translation and activation of Atgs, and apoptotic caspases cleave Atgs to render them nonfunctional [97, 98]. However, proapoptotic BH3-only proteins like Bax disrupt the antiautophagic role of Bcl-2/Bcl-xL, resulting in induction of autophagy [64, 101]. Similarly, proapoptotic Mtd/Bok is a powerful autophagy inducer by countering the antiautophagic role of Mcl-1 [103]. Autophagy is thought to be a prosurvival function, but overactivated autophagy accelerates apoptosis by excessively degrading cellular substances [66]. Moreover, Atgs are also said to provide the platform for activation of caspases [100]. In a state of OS, apoptosis and autophagy may occur simultaneously. They may be stimulated separately by OS and at the same time be induced by each other. Thus, their relationship may influence cell fate and lead to different pathophysiological outcomes.

Apoptosis and autophagy in human trophoblasts are interrelated pathways (Figure 3). Evidences showed that basal autophagy diminishes apoptosis in favor of cell survival, and apoptosis-associated proteins can induce autophagic protective function [95, 96]. For example, p53 activation seems to inhibit the activity of mammalian target of rapamycin (mTOR) and consequently promotes the activation of autophagy proteins [96]. However, the above phenomena are not absolute. Caspase-related cleavage of Atgs destroys autophagic machinery [97]. Cytoplasmic p53 is also proven to hinder autophagy although nuclear p53 induce autophagy [98]. Overactivated autophagy could accelerate the cascade of apoptosis, leading to cell death even without the presence of some apoptotic effectors [99, 100]. Antiapoptotic Bcl-2 family members and proapoptotic BH3-only proteins are known to participate in the suppression and induction of autophagy, respectively [64, 101]. Death-associated protein kinase (DAPK) may also phosphorylate Beclin1 and thus promote the dissociation of Beclin1 from Bcl-xL and the induction of autophagy [102]. Another study demonstrated that Mtd/Bok is a powerful inducer of autophagy; meanwhile, Mcl-1 functions as a repressor of autophagic pathways, but cleaved products of Mcl-1, specifically Mcl-1c157, can trigger autophagy [103]. In PE, an oxidative milieu, the imbalance of the Mtd/Bok-Mcl-1 system together with the accumulation of cleaved Mcl-1 isoforms may offer a possible explanation for the excessive autophagy as some evidence showed [103].

## 5. Therapies for OS-Related Pathology

**Figure 4:**
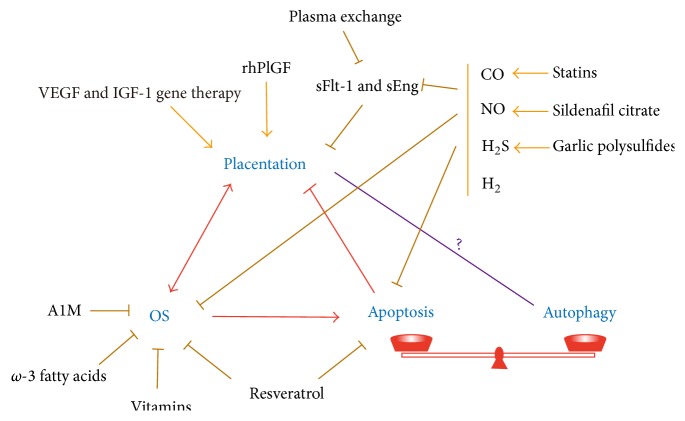
Therapies for OS-related disorders. Placentation and OS are closely interrelated. OS induced apoptosis may destroy normal placentation. At the same time, apoptosis and autophagy are maintained in a delicate balance, although the exact relationship between autophagy and placentation needs further verification. Chemicals or drugs with the potential to rescue pregnancy-related pathologies are listed. Some of them may have overlapping effects.

As we have already described, excessive OS is associated with the pathologies of spontaneous abortion, PE and IUGR. It seems that there are three ways to protect the placenta and fetus from OS attack and prevent or cure these disorders: reducing oxidative stress, promoting trophoblast invasion and angiogenesis, and suppressing apoptosis (Figure 4). As more mechanisms are discovered for autophagy in the placenta, adjustment of autophagy will also become a therapeutic approach.

### 5.1. Antioxidative Therapy

Antioxidants like vitamins C and E are often investigated as promising therapies to reduce OS during pregnancy and their beneficial effects have been shown in some trials [104, 105]. However, other investigators reached opposite conclusions; that is, vitamin C and E did not prevent PE or its complications in low-, moderate-, or high-risk women [106, 107]. Misuse of antioxidants seems to have harmful consequences that weaken trophoblast proliferation and even induce cell death [108]. The exact effects of, and optimum dosing and timing of, antioxidants use are still obscure and more investigations are required [108].

Hyperhomocysteinemia and NOS uncoupling are associated with increasing OS in pregnancy-related disorders like PE [109]. Resveratrol may be prospective to reverse the pathology by enhancing BH4 biosynthesis, reducing OS production, and increasing ROS scavenging [109]. BH4 supplementation can correct eNOS dysfunction [109]. A combined supplementation of folic acid, vitamin B_12_, and omega-3 fatty acids has also been proposed to counteract NOS uncoupling [110]. All the three nutrients are synergistically involved in the one-carbon cycle, restrain homocysteine-related OS, and promote methylation reactions [110]. Folic acid can also stimulate dihydrofolate reductase (DHFR) to reduce BH2 to BH4 [111]. Omega-3 polyunsaturated fatty acids are well-known anti-inflammatory and antioxidative nutrients [112]. According to the World Health Organization (WHO) and the Food and Agriculture Organization of the United Nations (FAO), a daily intake of omega-3 fatty acids is recommended during pregnancy [112]. These fatty acids also regulate placental vasculature by regulating the expression of proangiogenic genes through peroxisome proliferator activated receptors (PPAR) that are highly expressed in the trophoblastic layer [113].

Free fetal hemoglobin (HbF) and its metabolites heme and iron are highly reactive and proinflammatory molecules. In the process of PE, HbF induces damage in the placenta and even flows outside placenta to induce systemic OS [114–116]. *α*1-microglobulin (A1M) has displayed a protective effect against HbF induced damage in both placenta and kidney tissues in some experiments [114–116]. The protective mechanism of A1M was concluded to be as a reductase and antioxidant inducer that contributes to reorganization of the damaged collagen fibrillar structure [116]. It may also restore the angiogenic balance and has immunosuppressive effects [116].

N-acetylcysteine (NAC) and selenium supplementation are favourable ways to induce antioxidants as they participate in the formation of glutathione (GSH), glutathione peroxidase (GPx), and thioredoxin reductase (ThxRed) [117, 118]. Other investigations suggest that administration of melatonin, taurine, hypotaurine, lutein, and the dietary phytophenols curcumin, naringenin, and apigenin has potential preventive and therapeutic antioxidant effects to reduce infection and inflammation-induced OS and damage in the placenta [119–123].

### 5.2. Treatment in Promoting Placenta Formation

The therapeutic potential of some gas molecules like hydrogen (H_2_), NO, carbon monoxide (CO), and hydrogen sulfide (H_2_S) and their combined use in OS-related pregnancy disorders has drawn much attention [124]. They are proven to actively work to promote angiogenesis, regulate vasoactivity, and restrain inflammation, all of which stimulate normal placentation and provide healthy placental vasculature that avoids I/R caused injury.

The intake of H_2_-rich saline has been shown to protect maternal kidneys and placentas, as well as offspring, from oxidative injury [125, 126]. Clinical examination similarly suggested its benefit in maintaining vasculature health [127]. Besides oral ingestion, other therapeutic approaches include inhalation, injection, eye drops, baths, and cosmetics [125, 127, 128]. H_2_ shows satisfactory safety and strong practicability [125]. It has been used to treat decompression sickness in divers [125]. H_2_ also has mild effects without interfering with biological cell signaling, which is different from some conventional antioxidants [125, 128]. Additionally, the metabolic product of H_2_ is nontoxic water and overdose can be excreted via the lungs [125, 128]. Despite the extensive and varied effects, we do not know the specific targets and precise mechanism of H_2_ in these contexts [125, 128].

The conventional NO donors that have been studied in PE include glyceryl trinitrate, isosorbide dinitrate, and sodium nitroprusside [129]. An alternative, S-nitrosoglutathione (GSNO), however, has fewer side effects but greater anticoagulation effects without producing drug tolerance [129, 130]. N6022, a reversible inhibitor of GSNO reductase, has also been shown to improve endothelial function and to have an acceptable safety profile [129]. Aspirin has been used for its efficiency in reducing the incidence of PE in women with abnormal placentation [131]. Novel derivatives of aspirin, nitroaspirins, have been found to have a greater endothelial protective effect compared with aspirin [129]. Other novel NO-release compounds include diazeniumdiolates (NONOates) and dinitrosyl iron complexes with glutathione [129]. The cGMP specific phosphodiesterase inhibitor sildenafil citrate seems to strengthen the action of NO by inhibiting the downstream degradation of cGMP, second-messenger molecules that facilitate vasodilatation [132].

HO-1 (heme oxygenase-1)/CO is an endogenous pathway by which heme is oxidized to CO and biliverdin that inhibits sFlt-1 and sEng, as well as a positive regulator of VEGF-mediated vasculogenesis [133]. Statins are proven to induce HO-1 expression [133, 134]. The world's first randomized placebo-controlled trial, StAmP (Statins to Ameliorate Early Onset Pre-eclampsia), is underway and its outcome will reveal the feasibility of clinical use of statins in PE [135]. Moreover, edaravone and sofalcone have also been shown to potently activate antioxidant nuclear factor (erythroid-derived 2)-like 2/HO-1 and its related pathway [136–138].

H_2_S is required for healthy placental vasculature. Cystathionine *γ*-lyase (CSE) is the principal enzyme responsible for the endogenous production of H_2_S [139]. In many experiments, NaHS and GYY4137 are used as H_2_S donors [139, 140]. In clinical use, garlic-rich diets and garlic-derived organic polysulfides which include diallyl sulfide, diallyl disulfide, diallyl trisulfide, and ajoene aid the biological production of H_2_S [141].

Recombinant human PlGF may also be an attractive therapeutic strategy for its neovascularization capacity [142]. AMPK stimulator AICAR (5-aminoimidazole-4-carboxamide-3-ribonucleoside), which increases expression of VEGF, may be useful to restore angiogenic balance and mitigate renal and placental OS [143]. AMPK is also a signal transducer of autophagy, so its effect must be investigated further. Chemicals from plants, such as sodium tanshinone IIA sulfonate, pentoxifylline, and kraussianone-2, have been shown to improve uterine artery blood flow, resulting in improved fetal outcomes and decreased sFlt-1 and sEng in pregnant rats [144–146]. Plasma exchange by dextran sulfate apheresis may also be a therapeutic choice which can lower circulating sFlt-1, reduce proteinuria, and stabilize blood pressure without apparent harms to mother and fetus [147].

### 5.3. Antiapoptosis and Gene Therapies

Pomegranate juice is rich in the effective antioxidant punicalagin [148]. Both pomegranate juice and punicalagin are found to decrease OS and apoptosis in cultured STBs, characterized by decreased p53, downregulated HIF-1, and inactive caspases 9 and 3 [148]. The same antiapoptotic effects also seem to be induced by melatonin, CO, NO, resveratrol, and aspirin during I/R [149–153]. However, the mechanisms remain obscure. Potentially, the antiapoptotic role is mediated through the antioxidative effects of these compounds. Whether they independently restrain apoptotic signals remains to be elucidated. Maternal obstructive cholestasis during pregnancy also causes OS and apoptosis in rat placenta, which could be prevented by treatment with ursodeoxycholic acid [154]. The p38 MAPK inhibitor pamapimod may be an approach to reverse undesired apoptosis, but there are many safety problems when used during pregnancy [155].

VEGF gene therapy has shed new light on reversing impaired uteroplacental perfusion in complications like IUGR. Local delivery of the adenovirus-mediated VEGF gene during midgestation in IUGR sheep pregnancy models resulted in a distinct increased in fetal growth, enhanced placenta vasoactivity, and increased blood flow, without changing peripheral blood VEGF concentration [156]. Another study demonstrated a more restricted and safe response of uterine arteries to adenovirus-mediated VEGF-D^ΔNΔC^ gene therapy [157]. The underlying mechanism is believed to be a short-term upregulation of eNOS and endothelial cell proliferation, and in the long-term, more adventitial neovascularization is involved [157].

Placenta insulin-like growth factor-1 (IGF-1) also improved placental function and promoted fetal growth by inducing trophoblast proliferation, differentiation, migration, and survival when the gene was delivered to primary human placental fibroblasts [158]. Other researchers found the same effect following adenoviral delivery of IGF-1 into human trophoblast cells and murine models [159]. Moreover, IGF-1 seemed to facilitate placental amino acid transport [159]. This new discovery represents a new mechanism of IGF-1 in the restoration of fetal weight and rectification of placental insufficiency [159].

Individualized gene therapy is drawing increasing attention. It may benefit pregnant women who carry certain adverse gene variants. This method may avoid the side effects and drug tolerance found in conventional therapy and drive the development of much more specific and efficient drugs [160]. Further, the exploration of genetic polymorphisms and pathophysiological outcomes, together with the underlying molecular mechanisms, will lead to a deeper understanding of these disorders [160].

## 6. Conclusions

OS and placentation are closely interrelated. ROS/RNS is shown to influence placenta development while abnormal placentation may lead to OS and adverse consequences. Apoptosis and autophagy are homeostatic processes in trophoblast cells. Apoptosis and autophagy ensure normal cellular turnover, and undesirable apoptosis could also induce protective autophagy. Excessive autophagy can be discovered in some pathological complications. Whether a particular pathology is the result of excessive apoptosis or the independent action of dysfunctional autophagy is worth verifying.

In terms of the therapeutic methods we have discussed, many have produced promising results in animal models. Clinical trials are needed to confirm their effects in humans and to make sure that they are safe. The specific effects of autophagy in disorders of pregnancy may be a future area of emphasis and may be a useful treatment target in PE. Genetic therapy is a new direction for future medical development. Genes related to placentation are favorable targets. Individual therapy focusing on certain gene mutations will further improve efficacy. However, safe and reliable vectors, and optimized delivery techniques and times, must also be identified.

## Abbreviations

A1M: *α*1-microglobulin
ADP: Adenosine diphosphate
AICAR: 5-Aminoimidazole-4-carboxamide-3-ribonucleoside
AMP: Adenosine monophosphate
AMPK: AMP-activated protein kinase
Ang-1: Angiopoietin-1
APAF1: Apoptotic protease activating factor 1
ASK-1: Apoptosis signal-regulating kinase-1
Atg gene: Autophagy-related gene
ATM kinase: Ataxia-telangiectasia mutated kinase
Bcl-2: B-cell chronic lymphocytic leukemia/lymphoma 2
Bcl-xL: B-cell lymphoma-extra large
BH4: Tetrahydrobiopterin
BNIP-3: Bcl-2/adenovirus E1B 19 kDa interacting protein 3
CaMKK*β*: Calmodulin-dependent protein kinase kinase-*β*
CAP: Carboxyalkyl pyrrole
CMA: Chaperone-mediated autophagy
CO: Carbon monoxide
CSE: Cystathionine *γ*-lyase
CTB: Cytotrophoblast
eNOS: Endothelial nitric oxide synthase
DAPK: Death-associated protein kinase
DHFR: Dihydrofolate reductase
ERK: Extracellular regulated protein kinase
EVT: Extravillous trophoblast
FAO: Food and Agriculture Organization of the United Nations
FIP200: Focal adhesion kinase family-interacting protein of 200 kDa
FoxO: Forkhead box O
GPx: Glutathione peroxidase
GSH: Glutathione
GSNO: S-Nitrosoglutathione
H_2_: Hydrogen
H_2_O_2_: Hydrogen peroxide
H_2_S: Hydrogen sulfide
HbF: Fetal hemoglobin
hCG: Human chorionic gonadotrophin
HELLP syndrome: Hemolysis elevated liver enzymes and low platelets syndrome
HIF-1: Hypoxia inducible factor-1
HO^∙^: Hydroxyl radicals
HO-1: Heme oxygenase-1
hPL: Human placental lactogen
HSC70: Heat shock cognate protein 70
IGF-1: Insulin-like growth factor-1
IL: Interleukin
I/R: Ischemia/reperfusion
IUGR: Intrauterine growth restriction
JNK: Jun N-terminal kinase
LAMP-2A: Lysosome-associated membrane protein 2A
LC3: The microtubule-associated protein light chain 3
MAPK: Mitogen-activated protein kinase
Mcl-1: Myeloid cell leukemia factor-1
Mcl-1L: Myeloid cell leukemia factor-1 long isoform
Mcl-1S: Myeloid cell leukemia factor-1 short isoform
MCP-1: Monocyte chemotactic protein-1
Mdm2: Murine double minute 2
MHC: Major histocompatibility complex
MMP: Matrix metalloproteinase
Mtd/Bok: Matador/Bcl-2 ovarian killer
Mtd-L: Matador long isoform
mTOR: Mammalian target of rapamycin
MULE: Mcl-1 ubiquitin ligase E3
NAC: N-Acetylcysteine
NO: Nitric oxide
Nox: NADPH oxidase
NF-*κ*B: Nuclear factor *κ* B
O_2_^∙−^: Superoxide radicals
NONOate: Diazeniumdiolate
Nrf-2: Nuclear factor-erythroid 2 related factor 2
ONOO^−^: Peroxynitrite
OS: Oxidative stress
PARP-1: Poly(ADP-ribose) polymerase-1
PDK1: Phosphoinositide-dependent kinase 1
PE: Preeclampsia
PEA: Phosphatidylethanolamine
pGH: Placental growth hormone
PI3K: Phosphatidylinositol 3-kinase
PINK1: PTEN-induced putative protein kinase 1
PlGF: Placenta growth factor
RNS: Reactive nitrogen species
PPAR: Peroxisome proliferator activated receptor
PtdIns3K: Phosphatidylinositol 3-kinase
ROS: Reactive oxygen species
sEng: Soluble endoglin
sFlt-1: Soluble fms-like tyrosine kinase-1
SOD: Superoxide dismutase
StAmP: Statins to Ameliorate Early Onset Pre-eclampsia
STB: Syncytiotrophoblasts
TGF-*β*: Transforming growth factor-*β*
ThxRed: Thioredoxin reductase
TLR: Toll-like receptor
TSC1/2: Tuberous sclerosis complex 1/2
ULK1/2: Unc-51-like kinase 1/2
UVRAG: Ultra-violet irradiation resistance-associated gene
VEGF: Vascular endothelial growth factor
Vps34: Vacuolar protein sorting 34
WHO: World Health Organization
XO: Xanthine oxidase.
